# Microstructure Formations Resulting from Nanosecond and Picosecond Laser Irradiation of a Ti-Based Alloy under Controlled Atmospheric Conditions and Optimization of the Irradiation Process

**DOI:** 10.3390/mi15010005

**Published:** 2023-12-19

**Authors:** Dubravka Milovanović, Boris Rajčić, Dragan Ranković, Biljana Stankov, Miha Čekada, Jovan Ciganović, Dragica Đurđević-Milošević, Zoran Stević, Miroslav Kuzmanović, Tatjana Šibalija, Sanja Petronić

**Affiliations:** 1 Institute of General and Physical Chemistry, Studentski Trg 12/V, 11158 Belgrade, Serbia; 2 Faculty of Physical Chemistry, University of Belgrade, Studentski Trg 12-16, 11158 Belgrade, Serbia; 3 Institute of Physics, University of Belgrade, Pregrevica 118, 11080 Belgrade, Serbia; 4 Jožef Stefan Institute, Jamova cesta 39, 1000 Ljubljana, Slovenia; 5 Vinca Institute of Nuclear Sciences-National Institute of the Republic of Serbia, University of Belgrade, Mike Petrovića Alasa 12-14, 11351 Belgrade, Serbia; 6 School of Electrical Engineering, Technical Faculty in Bor, University of Belgrade, 11000 Belgrade, Serbia; 7 Faculty of Information Technology, Belgrade Metropolitan University, 11158 Belgrade, Serbia

**Keywords:** Ti6Al4V, picosecond laser, nanosecond laser, surface modification, argon-rich atmosphere, nitrogen-rich atmosphere, laser-induced periodic surface structures, laser-induced breakdown spectroscopy, Taguchi’s robust parameter design

## Abstract

This paper presents a study and comparison of surface effects induced by picosecond and nanosecond laser modification of a Ti6Al4V alloy surface under different ambient conditions: air and argon- and nitrogen-rich atmospheres. Detailed surface characterization was performed for all experimental conditions. Damage threshold fluences for picosecond and nanosecond laser irradiation in all three ambient conditions were determined. The observed surface features were a resolidified pool of molten material, craters, hydrodynamic effects and parallel periodic surface structures. Laser-induced periodic surface structures are formed by multi-mode-beam nanosecond laser action and picosecond laser action. Crown-like structures at crater rims are specific features for picosecond Nd:YAG laser action in argon-rich ambient conditions. Elemental analysis of the surfaces indicated nitride compound formation only in the nitrogen-rich ambient conditions. The constituents of the formed plasma were also investigated. Exploring the impact of process control parameters on output responses has been undertaken within the context of laser modification under different environmental conditions. Parametric optimization of the nanosecond laser modification was carried out by implementing an advanced method based on Taguchi’s parametric design and multivariate statistical techniques, and optimal settings are proposed for each atmosphere.

## 1. Introduction

Tremendous metal and metallic material consumption in contemporary everyday life and various branches of industry has resulted in metals like titanium and copper being considered critical raw materials. Titanium and its alloys are extensively used in aerospace, nuclear, marine and biomedical applications due to their superior physico-chemical properties, such as excellent corrosion resistance and biocompatibility, high specific strength, good ductility, and deformability [1,2]. This is the reason for continuous research interest focused on enabling prolonged usage of Ti-based materials by improving their wear resistance and biocompatibility, e.g., by protecting their surfaces [3].

Various techniques of surface modification for Ti-based alloys for improving their tribological properties, functionality and durability are being continuously investigated. Significant research interest is oriented towards selective surface hardening, spark plasma sintering methods, plasma nitriding and surface coating, as well as laser-based techniques, such as laser shock peening, laser deposition of coatings and thin films, pulsed laser remelting, laser hardening, laser surface texturing and laser patterning [4,5,6,7,8,9,10,11,12]. One of the challenges is achieving specific and selective modification, and this is the main advantage of laser-based techniques, due to laser beams’ high monochromaticity, coherence, directionality and intensity [13]. Using pulsed laser irradiation, from nanosecond to femtosecond pulse durations, enables accurate and effective enhancement of materials’ properties and functionality. Despite the complexity of the interaction of laser light with materials’ surfaces, this process is established as a highly controllable and precise way of producing surface modifications for various applications. The resulting effects, which vary from periodic surface structures like ripples, columns and craters in the macro-, micro- and nanometer ranges for the improvement of biocompatibility to structural coloration by modifying absorption properties or patterning to obtain antiadhesive surfaces and improve materials‘ physical properties, such as the microhardness of surfaces predisposed to additional stress, strongly depend on laser irradiation parameters (wavelength, pulse duration, pulse count, energy, frequency, beam mode, etc.) and materials’ properties (melting temperature, absorption coefficient, surface roughness, etc.) [12,14,15,16,17].

Besides the properties of the sample material and the laser irradiation properties, the resulting effects also depend on the environmental conditions. In recent years, materials’ modification with various assisted gases in controlled environments is gaining significant research attention [15,18,19,20,21] due to the possibility of protective-coating synthesis and/or suppressing the hydrodynamic effects that can affect modification precision. The environmental conditions in which the laser–material interaction takes place play an important role because many metallic alloys are prone to the formation of oxide layers on their surfaces that can affect their optical properties as well as increase the absorption of laser irradiation, but which can also lead to the formation of favorable compounds, such as nitrides, on the alloy surface, which can lead to improved chemical and mechanical properties in the material [15,18,19,20,21,22]. Specific qualities of laser irradiation have enabled its use as a rapid analytical tool for complex materials in the laser-induced breakdown spectroscopy (LIBS) technique [23]. Since a sample is ablated during LIBS, forming a plasma, it is significant to investigate the influence of the environmental conditions on LIBS qualitative analysis of Ti6Al4V [24].

Over the past few decades, extensive research efforts have been dedicated to studying the interaction between lasers and various materials across diverse optimization operations. The aim of optimizing irradiation conditions is to ensure the fulfilment of the desired, i.e., set, quality characteristics (i.e., process reactions). Various approaches have been implemented for laser material processing, including soft computing approaches [25,26,27] and statistical approaches [28,29]. The first approaches are based on neural models [27,30] that require a large amount of data that is not always possible to obtain experimentally. Another issue concerns the implementation of metaheuristic algorithms, such as particle swarm optimization (PSO) [25,26,27]. If the parameters of the algorithm are not properly selected, the algorithm converges to a local optimum [31], since its efficiency largely depends on the setting of its internal parameters. Statistical approaches are mainly based on the response surface method (RSM) [28], the desirability function approach [29] and the Taguchi method. Although the RSM is one of the most widely used methods for dealing with multiple responses, it does not directly incorporate response variation. The optimization method used in this study is based on Taguchi’s method and adequately solves the shortcomings of the aforementioned statistical methods.

In this study, picosecond and nanosecond laser modification of the titanium-based Ti6Al4V alloy surface under controlled environmental conditions is presented. The surface effects achieved by laser interaction in standard atmospheric conditions and argon- and nitrogen-rich atmospheres as well as a comparison of the resulting surface effects and nitriding possibilities are investigated and discussed. The influence of the different environmental conditions on spectral intensities in the LIBS analysis is also considered. The optimization of the laser parameters is carried out in standard atmospheric conditions and argon- and nitrogen-rich atmospheres in the nanosecond pulse regime.

## 2. Materials and Methods

The experiment involved the use of Ti6Al4V samples, each with dimensions of 60 × 60 × 2 mm. These samples were prepared by undergoing mechanical polishing and thorough cleaning prior to the laser irradiation process. The irradiation was conducted using nanosecond TEA CO_2_, nanosecond Nd:YAG, and picosecond Nd:YAG lasers under standard atmospheric conditions, which included an air atmosphere with a pressure of 1013 mbar and standard relative humidity. In addition to standard atmospheric conditions, the experiment also involved the use of nitrogen- and argon-rich atmospheres. These gases were supplied to the experimental setup through a nozzle connected to gas cylinders and equipped with a flow meter. The flow rate of these gases was maintained at approximately 1 L min^−1^. This setup enabled precise control of the environmental conditions during the laser irradiation of the Ti6Al4V samples. The parameters of the lasers and the conditions of laser irradiation are given in Table 1.

The effects of nanosecond TEA CO_2_, nanosecond Nd:YAG and picosecond Nd:YAG laser interactions with Ti6Al4V alloy under various environmental conditions were examined through a comprehensive analysis. An optical microscope Carl Zeiss EPITZP 2 (Jena, Germany) and scanning electron microscope JEOL JSM-6610LV SEM (Tokyo, Japan) coupled with an energy-dispersive spectrometer X-Max Large Area Analytical Silicon Drift EDS (Oxford, UK) were used to analyse changes on the alloy surface caused by lasers. For the surface topography characterization, the non-contact optical profilometer Zygo NewView 7100 (Middlefield, CT, USA) was used. Several surface parameters were examined via profilometry analysis: *Ra* is average surface roughness [32]; *rms* is the root-mean-square average of profile height deviations from the mean line [32]; *PV* is peak to valley [32]; and the ablation depth measurements were 2D profiles and 3D surface topography maps. The international standard ISO 25178 Surface Texture (Areal Roughness Measurement) was consulted for the filtering and evaluation processes [33]. The results obtained from profilometry analysis were processed in the Origin program (version 9.0). For the analysis of plasma generated in front of the sample target in different environmental conditions, the LIBS (laser-induced breakdown spectroscopy) technique was used. In general, plasma formed over the solid target emits spectral lines of elements found in the tested sample, and the intensities of these lines depend on the concentration of the emitting elements in the sample. In addition to the lines of elements from the target, lines of atoms that make up the atmosphere in which the plasma is induced by the laser will also be emitted in the plasma [34,35]. The spectrographs used in this work were the StellarNet LSR-UV2-14 and LSR-VIS4-14 (Tampa, FL, USA) with wavelength ranges of 200–400 nm and 400–600 nm, respectively.

Supplementary experiments for Nd:YAG nanosecond laser irradiation were conducted to further investigate the impact of various process control parameters on multiple output responses. These parameters included the number of accumulated laser pulses (*N_p_*) and the pulse energy (*E_p_*). The study focused on assessing the effects of these parameters on the following output responses: (1) *R_a_*; (2) *rms*; (3) *PV*; (4) *Kr*—presence of craters or crater formation (bulk material removal); (5) *DA*—depth of ablation; (6) *VA*—velocity of ablation (ablation rate, drilling depth per laser pulse); and (7) *C*—circularity (the ratio between the maximum and minimum diameters of the laser spot). These experiments were designed to identify the specific conditions that would result in the most precise modification and efficient ablation of the material, based on the surface characterization results obtained.

## 3. Results and Discussion

After laser irradiation of a material, several processes can be observed. Due to the high temperature of a laser beam, thermal melting occurs, as well as some plastic deformations of the material and, finally, cooling and resolidification. Also, depending on the pulse energy, crater formation and ablation may occur. Laser radiation primarily interacts with valence and conduction electrons, and the energy transfer time is determined by the laser pulse duration. In the case of very short pulses, the resulting distribution of energy to the electrons at the end of the pulse is not thermal [36,37,38,39]. Furthermore, the transferred (deposited) energy is redistributed to different energy states of the system; that is, the electrons transfer energy to the crystal lattice. The essential difference in the interaction of radiation of different lengths of impulse is presented more clearly when considering the timescale of the processes that are characteristic for those interactions [39]. Impulses with duration much longer than any process are considered to cause long energy relaxation in the system. This is the case for pulses in the nanosecond domain, as well as longer ones. Since all relaxation processes take place much faster than the duration of the impulse, the entire system is in an equilibrium state during the interaction [39]. When the laser pulses last longer than the lattice heating time, thermalization between the electronic subsystem and the lattice takes place during the pulse. In the nanosecond regime, the absorbed energy first heats the target surface to the melting point and then to the vaporization temperature. In the case of ablation with longer laser pulses, there is enough time for propagation of the heat wave in the target and a relatively large layer of molten material is formed. Evaporation occurs from the liquid metal, which significantly reduces the accuracy of laser metal processing. If micrometer-sized structures are preferred, sufficient precision can be achieved with nanosecond laser radiation. However, if it is necessary to form a structure of nanometer dimensions, it is preferable to use lasers with picosecond or femtosecond pulses. In the picosecond regime, the lattice temperature during the pulse remains much lower than the electron temperature. Balance is achieved only after the cessation of impulse. During the interaction of picosecond laser pulses with metals, energy transfer occurs for a few picoseconds, i.e., during the interaction. In this case, the material is confined in the area of the impact; it heats up at the end of the pulse, so theoretically there is no photon interaction with the melted or vaporized material, which results in more precise ablation.

**Figure 1:**
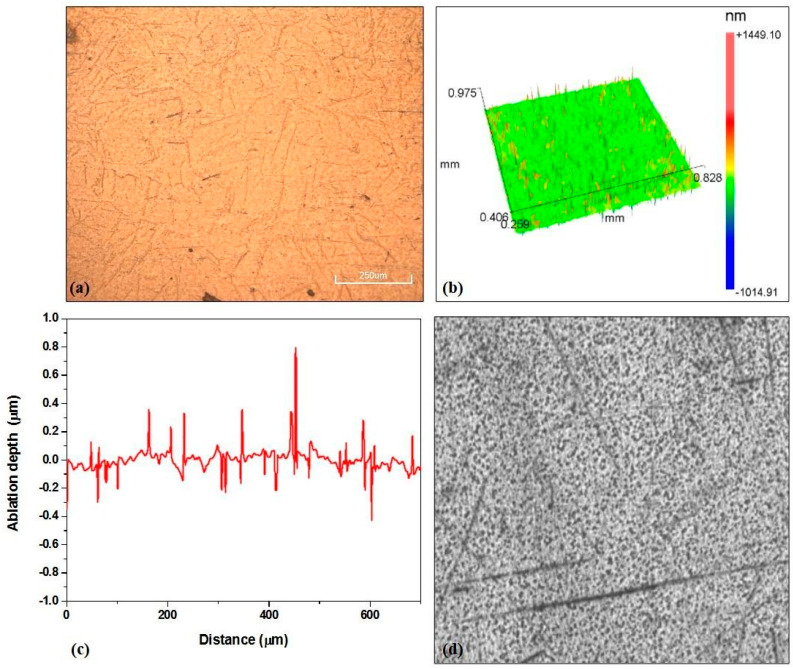
Unmodified surface of the Ti6Al4V alloy: (**a**) SEM microphotograph; (**b**) 3D map; (**c**) 2D profile; (**d**) intensity map.

Ti6Al4V is a titanium α + β alloy. In addition to the basic elements that make up the alloy, and depending on the application, oxygen may be present in the alloy, ranging from 0.08–0.4 wt%, where a high percentage of oxygen (0.4 wt%) plays the role of an enhancer of the alloy’s properties while a lower percentage of oxygen (with a lower percentage of nitrogen and aluminum) can improve characteristics such as ductility, corrosion resistance and resistance to crack growth [40]. The thermal conductivity of the alloy is 7.2 W m^−1^ K^−1^, the melting point is 1877–1941 K, and the thermal diffusivity is 0.022 cm^2^ s^−1^ [41]. Unmodified surface morphology and a two-dimensional profile are presented in Figure 1.

### 3.1. Determining Damage Threshold Fluence and Heat Affected Zone (HAZ)

**Figure 2:**
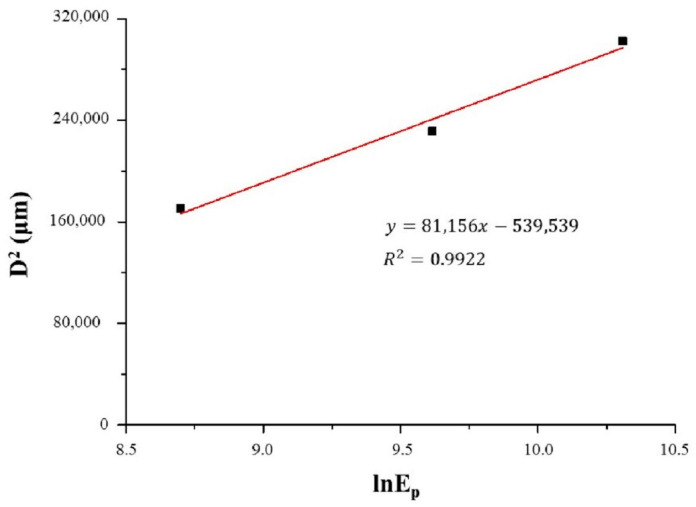
Example of the linear fit of *D*^2^ *= f* (*ln E_p_*) for 10 p, for Ti6Al4V irradiated by a laser beam of 150 ps pulse duration.

Damage thresholds, *F_th_*, which represent the minimum laser energy density value that causes visible changes on the target surface, were determined for Ti6Al4V alloy irradiated by picosecond and nanosecond laser radiation in the atmosphere, argon and nitrogen by the method proposed for Gaussian-shaped beams [42]. The threshold energy density values were determined by plotting the dependence of the diameter of the laser radiation trace in relation to the logarithmic value of the pulse energy [42]. From the linear fit slope and intercept, the value of the laser beam diameter, *ω_0_*, is obtained, and on the basis of this value the threshold fluence is calculated [42] (Figure 2). The obtained values *ω_0_* are 250.4 μm in air, 264.9 μm in a nitrogen-rich atmosphere and 201.4 μm in an argon-rich atmosphere, for a 150 ps laser pulse. The values of estimated *ω_0_* for a 5 ns laser pulse are 408.3 μm in air, 441.6 μm in a nitrogen-rich atmosphere and 433.2 μm in an argon-rich atmosphere.

However, this method is not suitable in the case of a multimode beam structure, as is the output beam of the TEA CO_2_ laser, so the threshold energy density values were estimated by direct observation of the target after each accumulated pulse with increasing pulse energy values until the first visible damage to the alloy surface was detected. The determined values of *F_th_* for Ti6Al4V in the present experimental conditions are given in Table 2. As expected, the inert nature of argon and nitrogen represses the laser/material interaction, and for 5 ns and 100 ns (long) laser pulse irradiation of the alloy surface, the *F_th_* value is lowest in air. However, for 150 ps (short) laser pulse irradiation, *F_th_* in air is higher by an average of 55 percent than in argon and nitrogen ambient conditions. This effect of argon and nitrogen environmental conditions is the opposite in the case of 5 ns and 100 ns (long) laser pulse interactions with the alloy surface, where the *F_th_* value is lowest in air, probably due to the 10 Hz laser repetition rate [43] and overall more intensive interaction, which caused more intensive plasma generation in front of the target and, subsequently, a plasma shielding effect in the air.

The effect that laser irradiation causes on the Ti6Al4V surface is determined by the amount of incident laser radiation absorbed by the material. For picosecond and nanosecond lasers, energy transfer from the laser beam to material is described by the heat effect zone, *HAZ*, which depends on the pulse duration and the thermal diffusivity of the material [44]:

(1) $$l_{th}=\sqrt{D\times \tau }$$

where *l_th_* (μm) is the length of thermal diffusion, i.e., *HAZ*, *D* (cm^2^ s^−1^) is thermal diffusivity, and *τ* (s) is pulse duration. Under the given experimental conditions, *HAZ* values are presented in Table 2.

The different morphological structures that form on the surface of the sample during the interaction with laser radiation also depend on the temperature reached on the surface of the material. For nanosecond laser radiation, estimation of the surface temperature value during laser/material interaction is done using the one-dimensional thermal conductivity equation [45]:

(2) $$∆T\approx \frac{\left(1-R\right)I_{o}\tau }{\rho C\sqrt{2D\tau }}$$

where *R* (%) is reflectivity (0.57% for TiAl4V for the wavelength of 1064 nm [46]), *I_o_* (W cm^−2^) is laser intensity, *C* (J kg^−1^ K^−1^) is thermal capacity (560 J kg^−1^ K^−1^ for Ti6Al4V [41]), ρ (g cm^−3^) is alloy density, *D* (cm^2^ s^−1^) is thermal diffusivity, and *τ* (s) is pulse duration.

For the interaction of nanosecond laser irradiation, under the present experimental conditions, the calculated temperatures were in the range of ~7000 K for 5 ns and ~15,000 K for 100 ns laser interaction. These temperatures, compared to the melting temperature of the Ti6Al4V alloy (1877–1941 K [41]), are more than sufficient for melting its surface and activating the gas above the surface, thus causing a reaction between the alloy sample and the surrounding gas. For picosecond laser interaction with the materials, the temperature model is more complex, and the two-dimensional equation has to be solved [45].

### 3.2. Surface Characterization

#### 3.2.1. Nanosecond Laser Irradiation—100 ns Pulse Duration, 10.6 μm Wavelength

**Figure 3:**
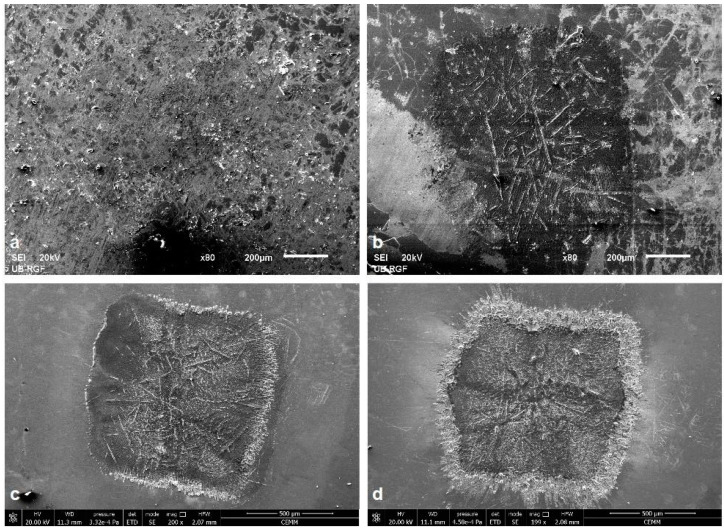
Ti6Al4V surface damaged areas after TEA CO_2_ laser action with (**a**) 50, (**b**) 400, (**c**) 800 and (**d**) 2000 pulses of 10.6 μm wavelength, 6.2 J cm^−2^ fluence and 100 ns pulse duration, in a nitrogen-rich atmosphere.

**Figure 4:**
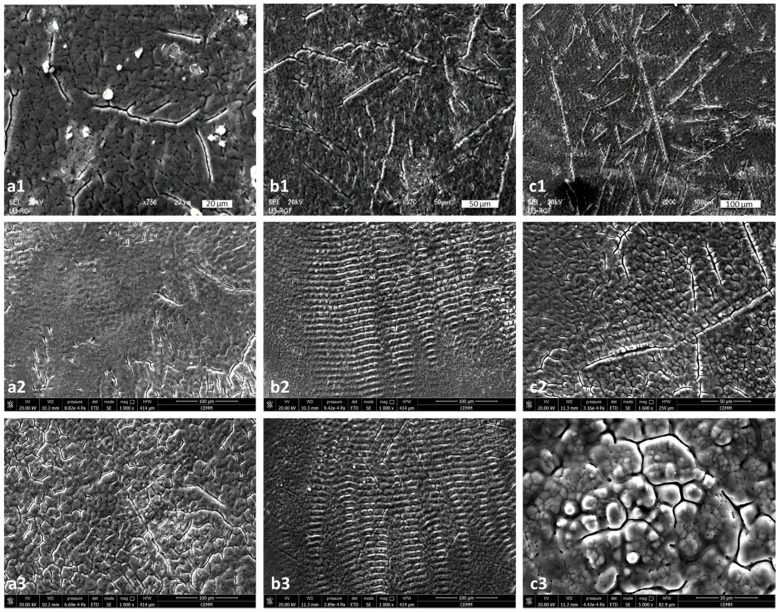
SEM microphotographs of the Ti6Al4V surface after TEA CO_2_ laser irradiation with a 100 ns pulse duration and 175 mJ pulse energy: (**a1**–**a3**)—air, fluence 5.3 J cm^−2^, (**b1**–**b3**)—argon-rich atmosphere, fluence 6.0 J cm^−2^, (**c1**–**c3**)—nitrogen-rich atmosphere, fluence 6.2 J cm^−2^; 1–400 pulses, 2–800 pulses and 3–2000 pulses.

Analysis of the morphological changes on the surface of the Ti6Al4V alloy after the effects of TEA CO_2_ nanosecond laser radiation at 155 and 175 mJ pulse energies and 6.0 J cm^−2^ and 6.2 J cm^−2^ fluences was subsequently performed using SEM. A multi-pulse mode of irradiation of the alloy sample was carried out, applying 50 to 2000 laser pulses. Although the values of the fluences are close, when analyzing the SEM microphotographs, the changes induced by 155 mJ pulses are not prominent, so the investigation focus is on the topography effects induced by laser action with 175 mJ pulse energy and 6.2 J cm^−2^ fluence (Figure 3 and Figure 4).

The TEA CO_2_ laser beam was in multi-mode; hence, the modification of the surface at the damaged areas is relatively homogeneously distributed and the spots are square-shaped (Figure 1). Irradiation of the surface with nanosecond laser radiation in all three ambient conditions leads to more prominent morphological changes on the surface of the alloy sample after 400 accumulated pulses (Figure 4(a1,b1,c1)) where material corrugation and cracking of the surface of the alloy followed by the appearance of microcracks, as well as the partial formation of granular structures probably matching the phase boundaries of the material, can be distinguished at the damaged areas.

With an increase in the number of applied pulses, the central and periphery can be distinguished, as can be seen after 800 and 2000 accumulated laser pulses (Figure 3c,d). There is melting of the alloy surface material as well as its cooling and hardening in the form of a “spike” near the periphery of the target, which is a consequence of the propagation of the molten material from the center to the periphery of the damaged area, as well as the occurrence of explosive ejection of the molten material or solidification of the front of the molten material from the center to the periphery. The center of the modified areas in air is characterized by resolidified molten areas and pronounced grain boundaries (Figure 4(a1–a3)) which is in accordance with expected thermal melting and subsequent cooling, without ablation and removal of material [15,21]. Also, the grains with pronounced boundaries and microcracks, due to rapid cooling, are noted at the center of the modified area after 800 and 2000 accumulated pulses in a nitrogen-rich atmosphere (Figure 4(c2,c3)) and, at the periphery of the modified area, there is a formation of periodic structures, but, due to excessive cracking of the alloy surface, they are of low intensity and period values could not be determined. However, modification in an argon-rich atmosphere leads to the formation of parallel laser-induced periodic surface structures (LIPSS) relatively uniformly over the modified area (Figure 4(b2,b3)) proportional to the laser wavelength of 10.6 μm, which are usually reported as the result of short (picosecond) and ultra-short (femtosecond) laser interactions with materials [47,48,49,50,51]. The periods of the LIPSS structures in the argon-rich atmosphere, close to the alloy irradiation wavelength (10.6 μm), are ~9.3 and ~11.0 μm for 800 and 2000 accumulated pulses, respectively. It can be concluded that low-frequency (LF) LIPSS structures are formed. There is not much reported research on LIPSS on Ti6Al4V in the nanosecond laser regime, because it is generally difficult to obtain such structures with long nanosecond pulses. LIPSS induced by TEA CO_2_ laser pulses have previously been reported on silicon [50]. On titanium, modified at a higher fluence value of 28 J cm^−2^, in an air and nitrogen-rich atmosphere, no LIPSS were reported [21]. Reported experiments with fiber laser multi-line scanning presented the formation of LIPSS only on a Ti6Al4V surface modified in nitrogen [15]. From our experiment, it can be concluded that the 10.6 μm pulse duration TEA CO_2_ multimode laser output at relatively low fluence values, approximately 6.0 J cm^−2^, could be used for patterning of the Ti6Al4V surface in argon- and nitrogen-rich ambient conditions. LIPSS formation is probably possible due to the low value of fluences and suppressed hydrodynamic effects under the influence of the inert nature of argon and nitrogen. It is important to note that, analyzing the SEM micrographs, no ablation, e.g., crater formation, was observed with the TEA CO_2_ nanosecond laser with multi-mode intensity distribution.

In the realm of structure formation in the molten phase and their subsequent transition into the solid phase through solidification, a noteworthy mechanism emerges when metals are subjected to laser irradiation at fluences near the threshold with a relatively low pulse count. Under these conditions, intriguing surface patterns manifest in the form of parallel waves that recur at intervals matching the wavelength of the incident laser. These distinctive patterns are commonly referred to as “laser-induced periodic surface structures” (LIPSS) [52,53]. The characteristics and properties of LIPSS are profoundly influenced not only by the number of laser pulses, often synonymous with the exposure time, but also by a range of other pivotal laser processing parameters. In accordance with the widely accepted plasmon combination theory, the formula used to determine LIPSS periods is expressed as:

(3) $$\Lambda_{LIPSS}=\frac{\lambda }{\eta \pm \sin\theta }$$

where *λ* is the laser wavelength, *η = R* [*ε_m_ε_d_*/(*ε_d_* + *ε_m_*)]^1/2^ is the real part of the effective refractive index for metal/air (~1), *R* is surface reflectivity, *ε_d_* is the dielectric constant, *ε_m_* is the complex dielectric constant of a metal, and *θ* is the incident angle [54]. It is evident from Equation (3) that the LIPSS period is proportional to the laser irradiation wavelength and, therefore, LIPSS formed as a result of TEA CO_2_ laser action should have values of ~10.6 μm, which corresponds to the obtained experimental values of LIPSS periods of 9.3 and 11.0 μm. In addition to the pulse number, LIPSS are influenced by various factors, including the laser wavelength, the angle at which the laser beam is incident, and the intrinsic properties of the material. Notably, LIPSS have demonstrated significant effects on altering the optical characteristics of metal surfaces, enhancing the active surface area, and improving biocompatibility [55].

#### 3.2.2. Nanosecond Laser Irradiation—5 ns Pulse Duration, 1064 nm Wavelength

**Figure 5:**
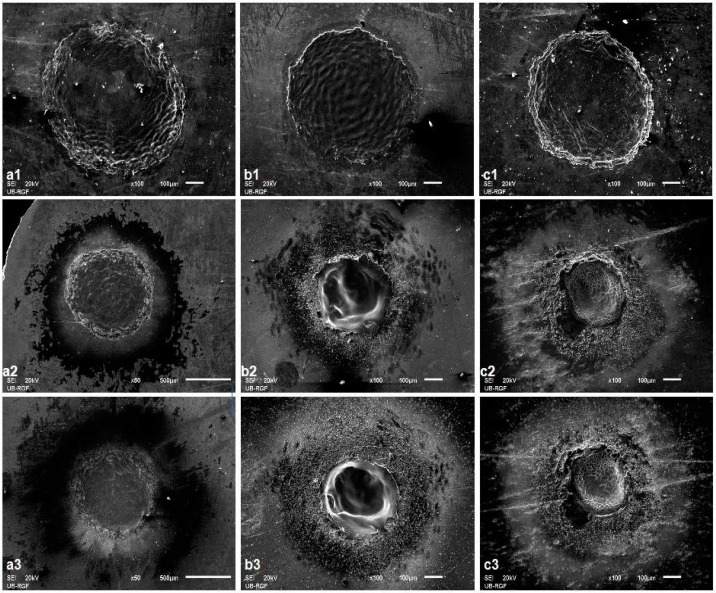
SEM microphotographs of the Ti6Al4V surface after 1064 nm Nd:YAG laser irradiation with a 5 ns pulse duration and 50 mJ pulse energy: (**a1**–**a3**)—air, fluence 19.1 J cm^−2^, (**b1**–**b3**)—argon-rich atmosphere, fluence 17.0 J cm^−2^, (**c1**–**c3**)—nitrogen-rich atmosphere, fluence 16.3 J cm^−2^; 1–10 pulses, 2–800 pulses and 3–2000 pulses.

The irradiation of the Ti6Al4V alloy with nanosecond laser radiation (Figure 5) is characterized by pronounced thermal effects on the surface, which is expected because the nanosecond pulses have a longer duration than the time required for relaxation processes on the target [37] and the Gaussian beam intensity distribution in the Nd:YAG laser system means that the intensity of the pulse is focused at the center and it decreases towards the periphery. Therefore, the obtained fluences for the same value of pulse energy are higher than with the multi-mode beam intensity distribution. Induced effects also include crater formation, which implies that material removal, e.g., ablation occurred and LIPSS were not observed under the present experimental conditions.

After irradiating the alloy with a single pulse with a pulse energy of 50 mJ and relatively low values of fluence (Table 1) the traces of the laser radiation spot are clearly distinguishable from material corrugation due to melting in all three ambient conditions. After 10 accumulated pulses, in addition to the more intense corrugation of the surface material in the center of the damaged area, the melting of the material caused the propulsion of the material and its movement towards the periphery of the target, Figure 5(a1,b1,c1). After 100 and more applied pulses, in an argon-rich atmosphere, at the higher energy density values of 57.7 and 84.9 J cm^−2^, the initial formation of a crater in the center of the modified target can be observed (Figure 5(a2,b2,c2)) as confirmed by the profilometry analysis, presented in Section 3.3. Profilometry analysis of the modified areas presented in Section 3.3 shows this trend continues after 800 accumulated pulses, now ocurring in air and nitrogen-rich atmospheres, but more dominant crater formation is again found in an argon-rich atmosphere, especially with a further increase in the applied pulses of 170 mJ energy and 57.7 J cm^−2^ fluence. With a further increase in the number of accumulated pulses, more pronounced hydrodynamic effects occur in the form of the formation of clearly defined crater edges in the form of solidified and pre-melted material, most pronounced in an argon-rich atmosphere (Figure 5(b2,b3)). The formation of granular structures on the periphery of the irradiated target is noticeable in atmospheres of argon, with a diameter of about 6.4 μm (Figure 5(b2,b3)) and nitrogen, with a diameter of about 4.3 μm, at lower pulse energy values (Figure 5(c2,c3)). The appearance of microcracks was observed with 100 accumulated pulses in a nitrogen-rich atmosphere and at an energy density value of 16.3 J cm^−2^. Crater formation is evident after applying a large number of pulses in all three ambient conditions, but as the crater inner walls in air and argon are relatively smooth, the craters formed in a nitrogen-rich atmosphere have a cone-like structure, distributed on the inner walls (Figure 5(c2,c3)). This is probably due to nitride formation, enabled by the optical breakdown of plasma in nitrogen, enabling chemical reactions of the surface material with the surrounding gas, as proposed in previous investigations [21].

#### 3.2.3. Picosecond Laser Irradiation—150 ps Pulse Duration, 1064 nm Wavelength

**Figure 6:**
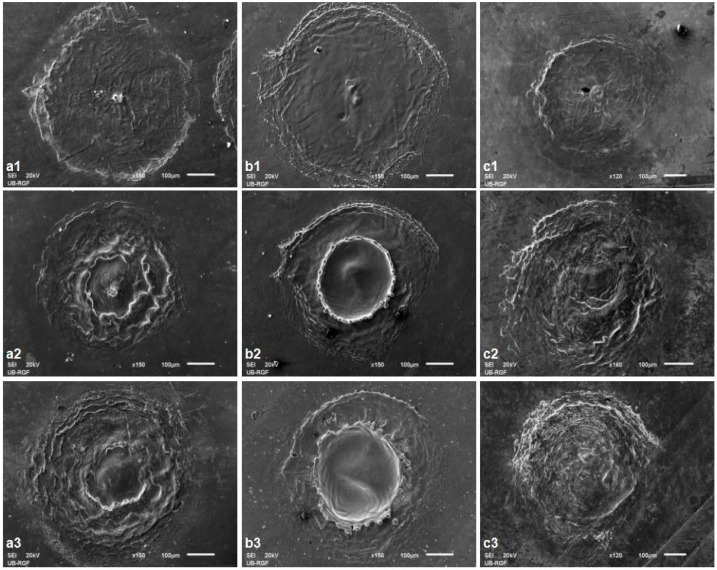
SEM microphotographs of the 1064 nm Ti6Al4V surface after Nd:YAG laser irradiation with a 150 ps pulse duration and 30 mJ pulse energy: (**a1**–**a3**)–air, fluence 30.5 J cm^−2^, (**b1**–**b3**)—argon-rich atmosphere, fluence 47.1 J cm^−2^, (**c1**–**c3**)—nitrogen-rich atmosphere, fluence 27.2 J cm^−2^; 1–10 pulses, 2–100 pulses and, 3–400 pulses.

**Figure 7:**
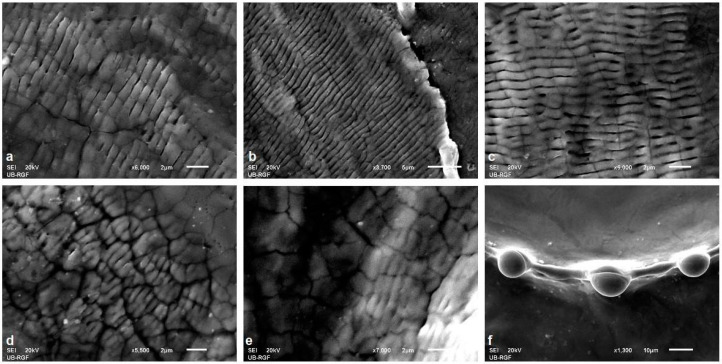
Periodic surface structures on a picosecond laser modified Ti6Al4V surface after (**a**) 400 pulses at 6.10 J cm^−2^ in air; (**b**) 200 pulses at 5.40 J cm^−2^ in a nitrogen-rich atmosphere; (**c**) 400 pulses at 23.5 J cm^−2^ in an argon-rich atmosphere, (**d**) 100 pulses at 15.2 J cm^−2^ in air, (**e**) 100 pulses at 3.6 J cm^−2^ in a nitrogen-rich atmosphere, (**f**) 100 pulses at 47.1 J cm^−2^ in an argon-rich atmosphere.

The effects of 150 ps laser irradiation on the Ti6Al4V alloy surface are prominent with an increasing pulse count from 10 to 400 and output energy ranging from 6 to 30 mJ in air and in argon- and nitrogen-rich atmospheres (Figure 6 and Figure 7). The main features are craters, increased roughness, microcracks and LIPSS. However, the morphological changes that arise significantly differ at different ambient conditions, with an increase in accumulated pulses (Figure 6). At a low pulse count of 10 pulses, there are small, uniformly distributed changes in topography, material corrugation and therefore surface roughness (Figure 6(a1,b1,c1)). An increase in the pulse count at constant pulse energy leads to prominent crater formation in the air and argon-rich atmospheres (Figure 6(a2,a3,b2,b3)). In nitrogen-rich atmosphere, a pool of resolidified molten material and corrugation occur, as well as shallow craters (Figure 6(c2,c3)). The reason for the absence of deeper craters may lie in the fact that the energy of the laser radiation is not sufficient to eject the molten material or its vaporization has already had dominant thermal effects, so the molten material is partially accumulated at the bottom of the crater. An interesting feature is noted after 100 accumulated pulses in an argon-rich atmosphere (Figure 6(b2)). The rim of the formed crater exhibits an organized, crown-like pattern, which is probably the result of hydrodynamic effects due to the propagation of the molten material.

At the higher values of pulse energies, there is mostly the absence of a smooth central part in the modified target. At an output energy of 15 mJ, after 50 accumulated pulses in air, microcracks are observed at the irradiated areas caused by the rapid cooling of the target surface after the end of the irradiation. These microcracks appeared at both the peripheral and central parts of the modified area. With an increase in the number of accumulated pulses, there is also the appearance of granular structures in the air at 30.5 J cm^−2^ fluence, which are more pronounced in the nitrogen-rich atmosphere at 13.6 J cm^−2^ after 200 applied laser pulses. The average diameters of these granular structures are 1.8 μm.

Picosecond laser irradiation of the Ti6Al4V alloy produced LIPSS structures at pulse energy values of 6 and 15 mJ, located on the periphery of the modified target. At a higher energy value of 30 mJ, no LIPSS structures were observed, probably due to the excessive energy of the pulse, which led only to intensive melting of the material both in the center and on the periphery of the modified target. Selected SEM microphotographs of LIPSS structures are shown in Figure 7a–e. The most pronounced LIPSS structures were formed in a nitrogen-rich atmosphere at 5.4 J cm^−2^ fluence with 200 accumulated pulses (Figure 7b). With a period value of approximately 900 nm, these can be classified as LF-LIPSS structures. After interaction with 100 accumulated pulses at 13.6 J cm^−2^ fluence in a nitrogen-rich atmosphere, LIPSS are observed relatively close to the center of the damaged area with a period value of ~570 nm, which is significantly below the value of the laser wavelength (Figure 7e). It can be observed that, with fluence increase, there is a decrease in the value of the period of LIPSS. In the air, structures are visible at the periphery of the modified target with period values of ~629 and 645 nm (HF-LIPSS structures) at pulse energy density values of 6.1 and 15.2 J cm^−2^, respectively, and for 100 and 200 accumulated pulses (Figure 7a–e). Regarding irradiation in the argon-rich atmosphere, LIPSS structures are only formed after 400 accumulated pulses and with an energy density value of 23.5 J cm^−2^. The period values of these structures are below the wavelength of laser radiation and are approximately 685 nm.

Besides the laser parameters (laser fluence, number of applied laser pulses, laser wavelength, polarization, incidence angle) and material properties (surface roughness, index of refraction, absorption coefficient, reflectivity, conductivity), LIPSS formation is influenced by environmental conditions, ambient pressure and molten material reorganization, if thermal effects occur during laser/material interaction. Generally, LIPSS period values show a decrease with an increasing number of accumulated pulses and/or increased laser fluence value, probably due to the fact that every subsequent pulse of the incident laser beam is deposited to the surface with altered properties, like increased roughness and specific surface [48,56,57]. The most interesting structures obtained in this experimental setting are the structures formed in an argon-rich atmosphere with an energy density value of 47.1 J cm^−2^ after 100 and 200 accumulated pulses (Figure 6(b2) and Figure 7f). Solidified drops of molten material are located at nearly equivalent distances of ~39 μm, forming a regular crown-shaped rim around the crater. The diameter of the resolidified droplet is ~10 μm. The proposed explanation is that the thermocavitation instability of the molten material on the surface can occur in the form of a micrometer-sized crown on the surface, and, as a result of the action of a larger number of laser pulses, energy density values are close to the ablation threshold density value [58]. It is likely that these structures form due to thermodynamic instability. The formation of structures of this type has been reported on thin metal films deposited on silicon and on the surface of silicon after the action of femtosecond laser radiation, with nanometer dimensions, under standard experimental conditions [58,59]. The formation of such structures after the action of picosecond laser radiation has not been reported in the studied literature.

### 3.3. Surface Roughness Investigation

**Figure 8:**
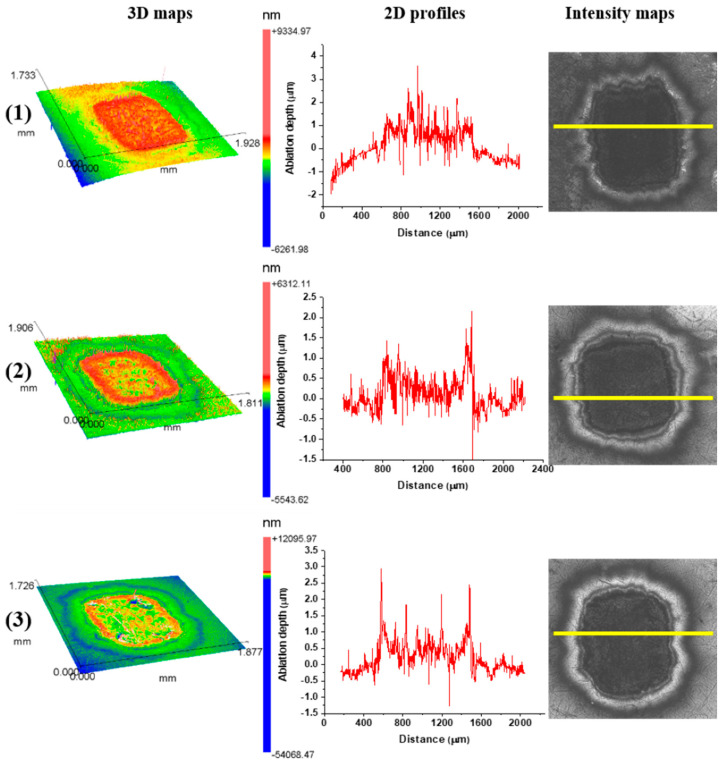
Cross-sectional profiles, three-dimensional representations and intensity maps of the Ti6Al4V surface after laser irradiation at 10.6 μm, pulse duration 100 ns, pulse energy 175 mJ and pulse count 400 in (**1**) air and (**2**) argon-rich and (**3**) nitrogen-rich ambient conditions. Yellow lines in the intensity maps indicate cross-section lines of the 2d profiles.

**Figure 9:**
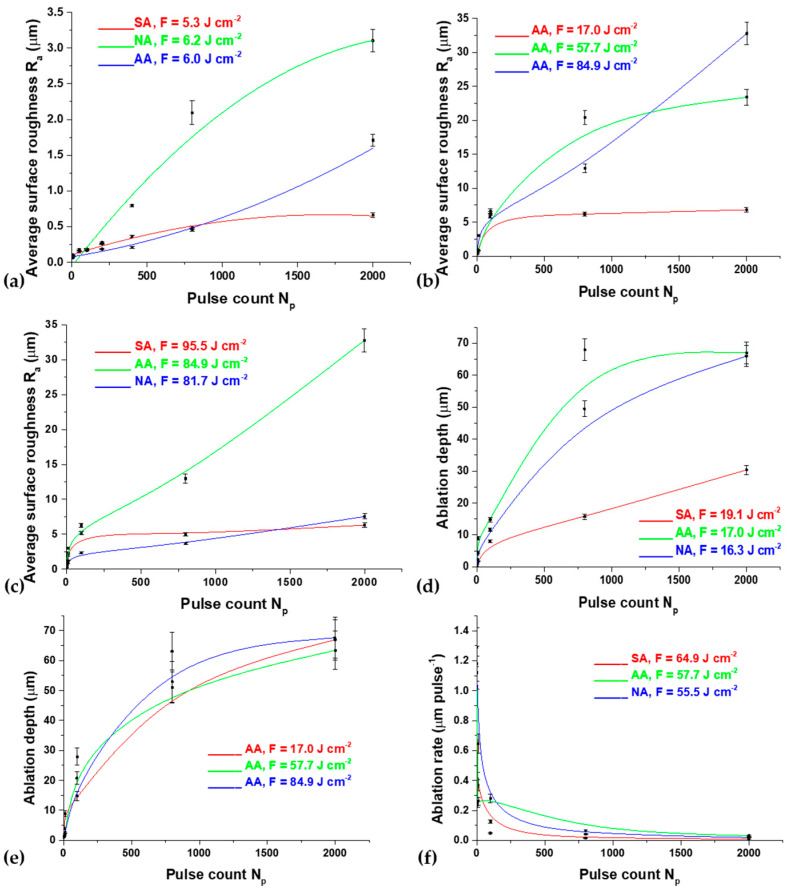
Nanosecond irradiation of Ti6Al4V—diagrams of surface parameters’ dependence on pulse count, *N_p_*: (**a**) average surface roughness, *R_a_*, at 175 mJ energy, 10.6 μm wavelength and 100 ns pulse duration; (**b**) average surface roughness, *R_a_*, at 250 mJ energy, 1064 nm wavelength and 5 ns pulse duration; (**c**) average surface roughness, *R_a_*, at 1064 nm wavelength and 5 ns pulse duration with different pulse energies in an argon-rich atmosphere; (**d**) ablation depth at 50 mJ energy, 1064 nm wavelength and 5 ns pulse duration; (**e**) ablation depth at 1064 nm wavelength and 5 ns pulse duration with different fluences in an argon-rich atmosphere and (**f**) ablation rate at 170 mJ pulse energy, 1064 nm wavelength and 5 ns pulse duration.

**Figure 10:**
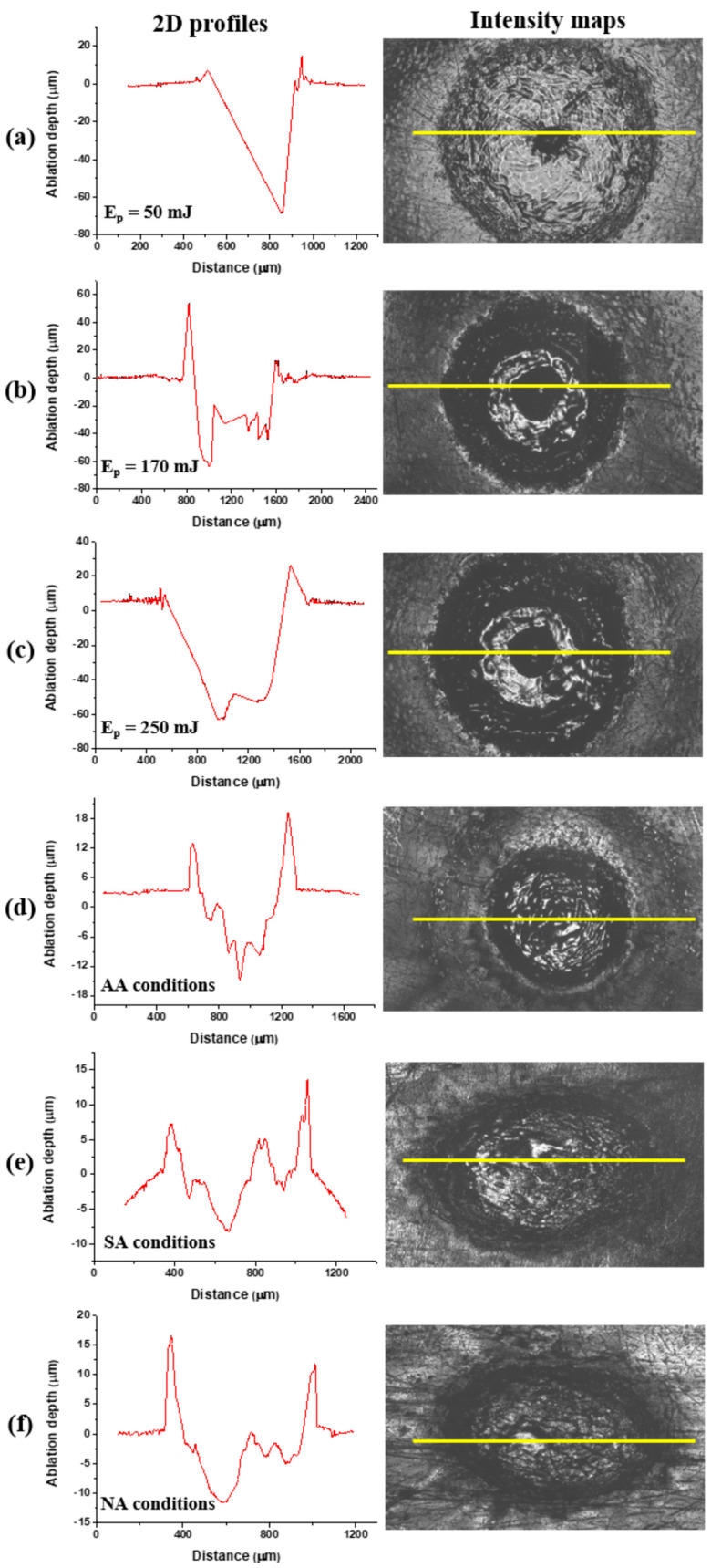
Cross-sectional profiles and intensity maps of the Ti6Al4V surface after laser irradiation at 1064 nm wavelength, pulse duration 5 ns and (**a**–**c**) *N_p_* = 800, argon-rich atmosphere; (**d**–**f**) *N_p_* = 100, *E_p_* = 50 mJ. Yellow lines in the intensity maps indicate the cross-section lines of the 2d profiles.

**Figure 11:**
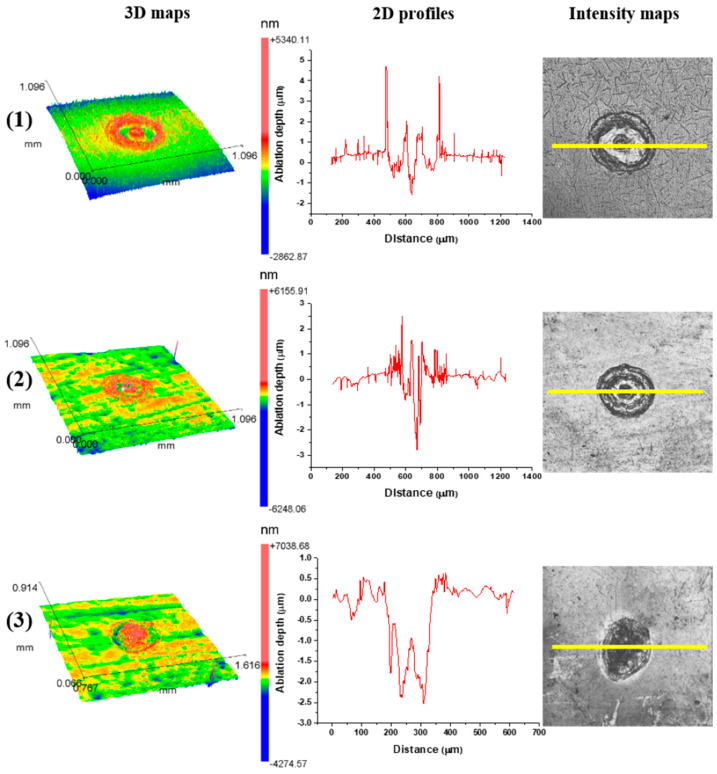
Cross-sectional profiles, three-dimensional representations and intensity maps of the Ti6Al4V surface after laser irradiation at 1064 nm, pulse duration 150 ps, pulse energy 6 mJ and pulse count 10 in (**1**) air and (**2**) argon-rich and (**3**) nitrogen-rich ambient conditions. Yellow lines in the intensity maps indicate the cross-section lines of the 2d profiles.

**Figure 12:**
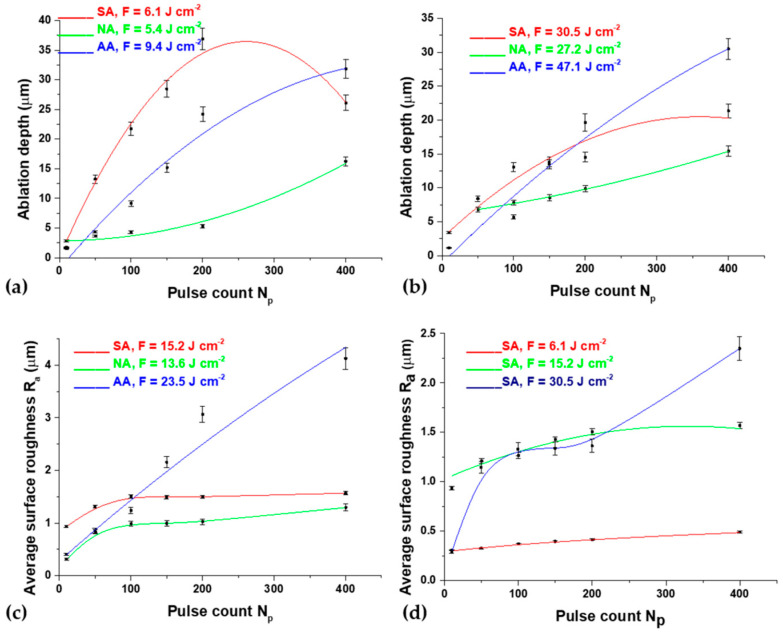
Picosecond irradiation of Ti6Al4V at a 1064 nm wavelength and 150 ps pulse duration—diagrams of surface parameters’ dependence on pulse count, *N_p_*, in different experimental conditions: (**a**) ablation depth at 6 mJ pulse energy, (**b**) ablation depth at 30 mJ pulse energy, (**c**) average surface roughness, *R_a_*, at 15 mJ pulse energy and (**d**) average surface roughness, *R_a_*, at different fluences, in standard atmospheric conditions.

After TEA CO_2_ laser irradiation, at a 10.6 μm wavelength and 100 ns pulse duration, pronounced corrugation and no crater formation is evident in all ambient conditions. Examination of the two-dimensional profiles and three-dimensional maps of the Ti6Al4V surface confirmed the accumulation and redistribution of the molten and cooled material and no crater formation (Figure 8). Nd:YAG laser action at a 1064 nm wavelength, 5 ns pulse duration and output energy of 50 mJ caused ablation and crater formation on the alloy surface (Figure 9d,e and Figure 10d–f). A Ti6Al4V area modified by Nd:YAG picosecond laser irradiation of 10 pulse at a 6 mJ output energy is characterized by shallow craters and increased surface roughness (Figure 11 and Figure 12).

The dependence of the average surface roughness, *R_a_*, on accumulated pulse number, pulse count *N_p_*, is given in Figure 9a. The value of *R_a_* ranges from ~0.2–0.3 µm for 200 pulses to ~1.3 µm for 2000 pulses for 155 mJ pulse energy. At a pulse energy value of 175 mJ, up to 200 accumulated pulses, *R_a_* is about 0.25 µm, while, after 400 pulses, an increase in the roughness value is again noticeable (Figure 9a). In air, *R_a_* increases up to ~0.75 µm for *N_p_* = 2000 in an argon-rich atmosphere, up to ~1.4 µm for *N_p_* = 2000, and, in a nitrogen-rich atmosphere, the highest value of the *R_a_* parameter is ~3.25 µm for *N_p_* = 800. The biggest increase in roughness is in the presence of nitrogen, probably due to the formation of cone-like structures, i.e., nitrides, which were detected by SEM. Under the given experimental conditions, it can be concluded that the ablation efficiency is low, but, considering the clear formation of LIPSS structures (Figure 3) as well as the good values of the surface roughness parameter, this alloy irradiation is suitable for patterning the alloy surface.

Considering the variety of morphological changes at the surface of the Ti6Al4V alloy induced by nanosecond laser radiation of a 1064 nm wavelength and 5 ns pulse duration, profilometric analysis provided more detailed information regarding surface profile, topography, ablation depth, ablation rate and surface roughness (Figure 9b–f).

By plotting the dependence of *R_a_* in relation to the number of accumulated pulses, it can be concluded that at *N_p_* ≤ 10, *R_a_* does not exceed the value of ~3 μm. By increasing the pulse count up to 2000 accumulated pulses at the lowest fluences, the *R_a_* values in air at 19.1 J cm^−2^ fluence and a nitrogen-rich atmosphere at 16.3 J cm^−2^ fluence are similar and amount to ~13 μm, while, in an argon-rich atmosphere at 17.0 J cm^−2^ fluence, *R_a_* amounts to ~6.8 μm. Roughness is more pronounced at higher fluences, where the *R_a_* value of ~32.8 μm is the highest in the argon-rich atmosphere at 84.9 J cm^−2^ fluence. In general, in the presented experimental conditions, the surface roughness is similar in air and a nitrogen-rich atmosphere as opposed to an argon-rich atmosphere. Again, it is concluded that the argon-rich atmosphere, at 17.0 J cm^−2^ fluence, is the most suitable for the controlled production of a clearly defined crater on the surface of the Ti6Al4V alloy, considering that, at this fluence, the average surface roughness of the alloy in the argon-rich atmosphere has the lowest value. This can be confirmed by the diagram in Figure 9c, where the value of the *R_a_* parameter does not increase but reaches saturation after 10 accumulated pulses.

The dependence of the ablation depth in relation to the number of accumulated pulses in different ambient conditions under the action of a laser beam of energy 50 mJ is presented in Figure 9d. At a pulse count below 10, the ablation depth is relatively low with an average value of ~2–4 μm in air and nitrogen-rich atmospheres. In an argon-rich atmosphere at 17.0 J cm^−2^ fluence with 10 accumulated pulses, there is an increase in the value of the ablation depth up to ~9 μm (Figure 9d). After 10 accumulated pulses, at 84.9 J cm^−2^ fluence, the value of the ablation depth significantly increases and reaches values up to ~67.6 μm with 2000 accumulated pulses (Figure 9e) in an argon-rich atmosphere, which is also verified by the cross-section profile in Figure 10c. In air, the lowest values of ablation depth are at the lowest values of pulse energy density, 19.1 J cm^−2^ and 64.9 J cm^−2^, while, at the highest value of energy density of 95.5 J cm^−2^, the ablation depth is similar to that in a nitrogen-rich atmosphere and is ~30 μm. After 2000 accumulated pulses, the ablation depth values generally reach saturation (Figure 9d). It can be noted that effective ablation of the Ti6Al4V alloy surface is achieved after nanosecond laser irradiation with a wavelength of 1064 nm and a pulse duration of 5 ns at all investigated fluences in an argon-rich atmosphere (Figure 9e).

The dependence of ablation speed on an increase in the applied pulses with 170 mJ energy under different ambient conditions is shown in Figure 9f. With an increase in the number of accumulated pulses, the values of the ablation rates drop sharply to *N_p_* = 100. In an argon-rich atmosphere, at lower values of pulse energy density and at a small number of accumulated pulses, the ablation rate has the highest value with a 2.37 μm pulse^−1^. That value decreases by a factor of three after 10 accumulated pulses. In air and a nitrogen-rich atmosphere, the values of ablation rates at a low number of accumulated pulses are, on average, 0.25–0.40 μm pulse^−1^ and decrease with an increase in the number of applied pulses by a factor of 2–3. At higher values of pulse energy density, in air and an argon-rich atmosphere, ablation rate values are similar and, on average, 1.10–1.29 μm pulse^−1^ (Figure 9f). With *N_p_* > 100, the values of the ablation rates reach saturation below ~0.03 μm pulse^−1^. These results are in agreement with the results for the value of the ablation depth and with the SEM analysis, so it can be concluded that, under the presented experimental conditions, the ablation effect for Ti6Al4V alloy is the most dominant in an argon-rich atmosphere.

The dependence of the ablation depth achieved on the surface of the Ti6Al4V alloy by 150 ps laser pulses at a 1064 nm wavelength is presented in Figure 11 and Figure 12a,b. It is notable that the highest ablation depth value, about 36 μm, is obtained in the air at 6.1 J cm^−2^ fluence and 200 accumulated pulses (Figure 12a). In general, the average lowest values of ablation depth are obtained in the nitrogen-rich atmosphere (Figure 12a,b). The highest achieved depth value in the nitrogen-rich atmosphere is about 15 μm at an energy density value of 5.4 J cm^−2^ with 400 accumulated pulses (Figure 12a). With a pulse count up to 50, the average value of the ablation depth is ~5 μm, and the value of the ablation depth increases with a further increase in pulse count. From 50 to 200 accumulated pulses, the tendency of the ablation depth value to increase is prominent, especially in air and argon-rich atmospheres. After 400 accumulated pulses, the values of ablation depth either decrease or saturate, probably due to excessive melting of the material inside the target and its redistribution, e.g., the inability to expel the molten material from the surface of the modified areas (Figure 12a). In air, the maximum value of ablation depth at a 200 pulse count decreases to ~25 μm at a 400 pulse count. However, from a comparison of the SEM and the ablation depth values, it can be concluded that the most ideal crater formation is achieved in an argon-rich atmosphere.

After picosecond laser action in an argon-rich atmosphere, the highest value of average surface roughness *R_a_*, 4.13 μm, is achieved with 400 accumulated pulses at 23.5 J cm^−2^ fluence (Figure 12c). This is in agreement with SEM, where it is noted that the most pronounced surface effects are obtained in an argon-rich atmosphere. Up to 100 accumulated pulses, *R_a_* values are relatively low and amount to ~1.5 μm (Figure 12c). After increasing pulse counts up to 400, at 13.6 J cm^−2^ fluence, the lowest *R_a_* value, 1.98 μm, is obtained in a nitrogen-rich atmosphere, and the highest value is achieved in an argon-rich atmosphere. The dependence of *R_a_* on the pulse count at different fluences in air is presented in Figure 12d. Modification in air up to 100 accumulated laser pulses at lower fluences is characterized by a slight increase in the *R_a_* value and a prominent increase in *R_a_* at 30.5 J cm^−2^ fluence. With a further increase in the pulse count at 6.1 and 15.2 J cm^−2^ fluence values, *R_a_* subsequently reaches a plateau at 0.5 and 1.5 μm due to the flattening of the molten pool of material, but, after 200 pulses at 30.5 J cm^−2^ fluence, average surface roughness increases up to 2.4 μm.

### 3.4. Chemical Analysis

Estimation of changes in elemental composition influenced by TEA CO_2_ nanosecond and Nd:YAG picosecond laser interaction with the Ti6Al4V surface under three different ambient conditions was performed with EDS and LIBS techniques.

**Figure 13:**
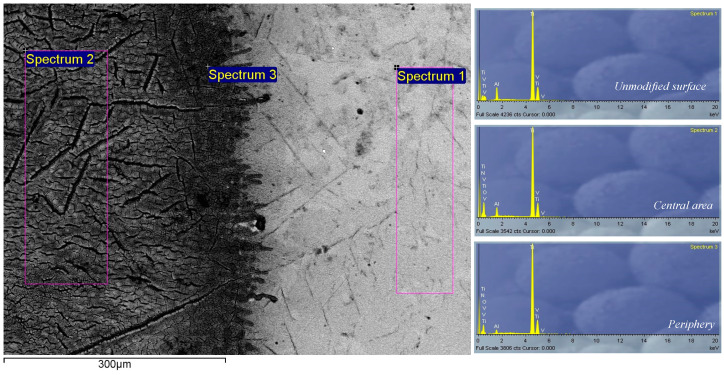
Locations and EDS spectra of unmodified and nanosecond-laser-modified Ti6Al4V surface in a nitrogen-rich atmosphere; *E_p_* = 175 mJ, *N_p_* = 2000. Locations of EDS-recorded spectra: spectrum 1—unmodified surface; spectrum 2—central area; spectrum 3—periphery.

The EDS spectrum of the alloy before modification, selected spectra after nanosecond laser irradiation of the surface and corresponding measurement locations are presented in Figure 13. EDS analysis was performed at several locations on each irradiated target, including the center and periphery of the target, and the results are presented as weight percentages of the elements (Table 3).

In air, at 3.9 J cm^−2^ fluence, there is an expected detection of Ti, Al and V, which represent constituents of the Ti6Al4 alloy, but also the detection of oxygen, which indicates the formation of oxides on the surface of the alloy. However, in a nitrogen-rich atmosphere, at 6.2 J cm^−2^ fluence, a much larger number of applied pulses is needed to form nitride compounds, so, at 400 accumulated pulses, no nitrogen is detected, while, at 2000 pulses, nitrogen appears both in the center and on the periphery of the modified target, at 12.63 and 9.89 weight percent, respectively (Table 3). In an argon-rich atmosphere, at 6.2 J cm^−2^ fluence, oxygen was detected in the amounts of 35.04 and 38.14 percent by weight in the center of the target and on the periphery, respectively (Table 3).

**Figure 14:**
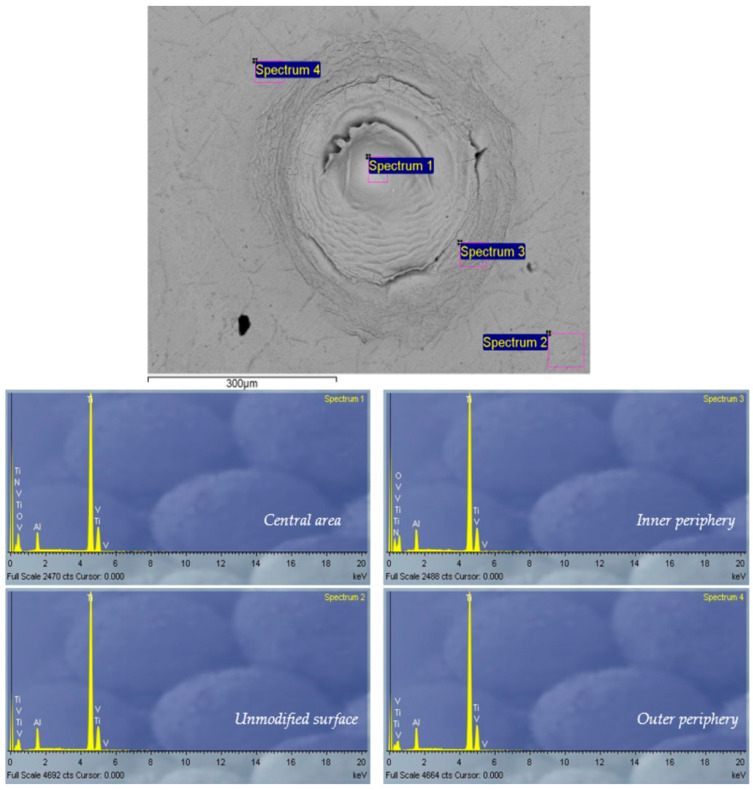
Locations and EDS spectra of unmodified and picosecond-laser-modified Ti6Al4V surface in a nitrogen-rich atmosphere; *E_p_* = 15 mJ, *N_p_* = 100. Locations of EDS-recorded spectra: spectrum 1—central area; spectrum 2—unmodified surface; spectrum 3—inner periphery; spectrum 4—outer periphery.

The EDS spectrum of the alloy before modification, selected spectra after picosecond laser irradiation of the surface and corresponding measurement locations are presented in Figure 14. EDS analysis was performed at several locations on each irradiated target, including the center and periphery of the target, and the results are presented as weight percentages of the elements (Table 4). At a pulse energy of 15 mJ, in air, irradiation of the alloy leads to oxidation of its surface, in the center as well as on the periphery of the modified area (Table 4).

As the amount of oxygen increased on the surface, the amounts of titanium, aluminum, and vanadium decreased. Given the higher reactivity of titanium compared to aluminum and vanadium, titanium oxides were probably the first to be formed. These oxides have a significant impact on applications in biomedicine in terms of the longevity of an implant because it is known that titanium oxides increase resistance to corrosion [60]. Also, they influence the bioactivity of titanium implants as well as the role of the central layer in combination with hydroxyapatite, exhibiting osteoinductive properties [60].

Regarding the irradiation of the Ti6Al4V alloy in a nitrogen-rich atmosphere, it is evident that the formation of nitride compounds occurs. After irradiation at every pulse energy value, EDS surface analysis detected nitrogen in the range of an average of 10 wt% at the center and the periphery of the spot area (Table 4), even though its presence could be expected as the result of laser interaction with the alloy surface in air and in an argon-rich atmosphere. Oxygen is detected on the surface of each irradiated target, but in a smaller percentage due to the increase in nitrogen content. Regarding the irradiation of the Ti6Al4V alloy in an argon-rich atmosphere, no significant changes occur in terms of the formation of compounds other than oxides. The share of oxygen ranges up to ~20 wt% at each pulse energy value (Table 4).

**Figure 15:**
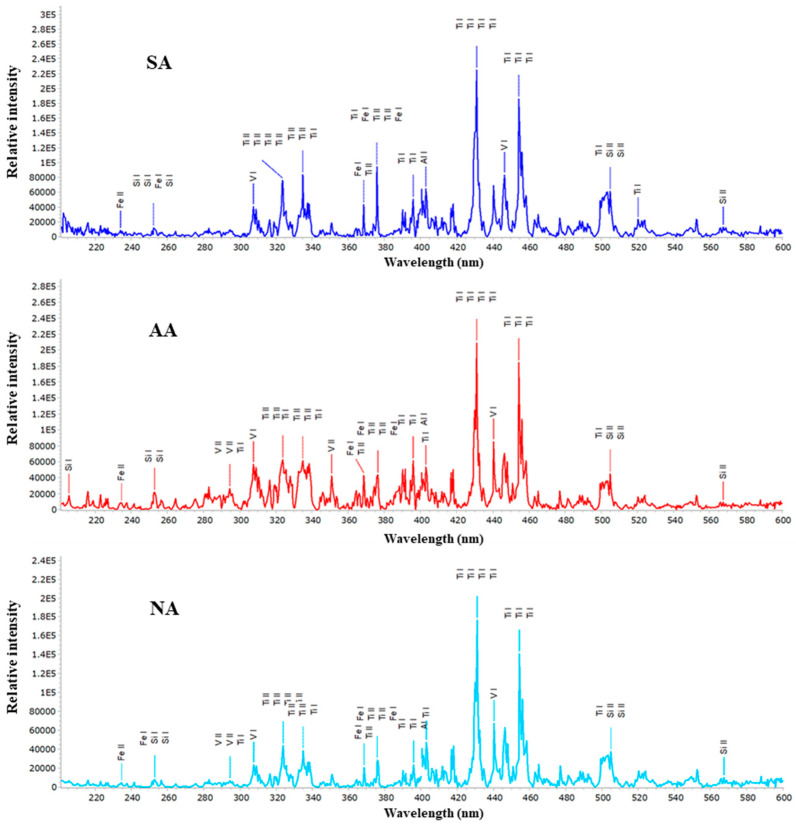
LIBS spectra of the Ti6Al4V alloy obtained by irradiation with a picosecond Nd:YAG laser in different atmospheres at atmospheric pressure.

Analysis of the plasma formed during the interaction of the picosecond laser beam with the Ti6Al4V surface was performed using LIBS. The spectra for the Ti6Al4V alloy obtained after irradiation with a picosecond Nd:YAG laser with air, argon and nitrogen as the surrounding gases are presented in Figure 15. It can be concluded that the induced plasma is hot and dense due to the dominance of the ion lines of the constituent elements of the alloy. As for the emission of elements of the surrounding atmosphere, in all the presented results, the spectral lines of the elements are practically absent, which could indicate that the experimental conditions, primarily the position of the laser focus in relation to the target, favored the emission of metal elements from the sample target.

The preferred surface feature resulting from laser/material interaction depends on the intended application of the material. For example, the modification can be (i) crater formation, favorable for lubricating the surface to reduce friction; (ii) nitride/oxide formation as a protective layer; or (iii) formation of periodic surface structures and increased roughness, which can increase the biocompatibility of an implant material’s surface.

### 3.5. Process Optimization for Nanosecond Laser Modification

Exploring the impact of process control parameters on output responses has been undertaken within the context of laser modification (1064 nm wavelength, 5 ns pulse duration) under different environmental conditions, which have been determined to yield the most precise modification and efficient ablation. This determination is based on the analysis provided in this paper (Section 3.2 and Section 3.3). Table 5, Table 6 and Table 7 display the experimental outcomes obtained under different atmospheres. Each response was subjected to three measurements (resulting in a sample size of three), and the tables present the average values derived from these measurements.

The optimization procedure is motivated by the quality loss (QL) function, proposed by G. Taguchi, which stipulates the user’s dissatisfaction when utilizing a product whose feature (response) diverges from the target value. This consideration becomes more complex when a product is defined by multiple responses, so the relative significance of each response for the users can be expressed by its QL value, without imposing any subjective weights. QL estimation implies the signal-to-noise ratio, simultaneously including both the response mean and the variation. Three types of responses are suggested by G. Taguchi [61]: nominally the best (NTB), whose goal is to reach a nominal (target) value; the smaller the better (STB), aiming to minimize the response value; and the larger the better (LTB), focused on maximizing the response value.

For the considered process, the response *Kr* belongs to the NTB type, and the remaining ones belong to the LTB type. Depending on the application, for instance, the micro-lubrication of tools, the goal value for response crater occurrence (*Kr*) is a nominal value of 1 and a circularity (*C*) value near 1, which means that a circular-shaped crater is formed. In the case of micro-patterning for increased biocompatibility or a more developed surface, responses related to surface roughness, such as *R_a_*, *rms*, and *PV* should be as high as possible. The computed QL values for the seven responses are normalized (NQL ∈ [0, 1]) (Table 8, Table 9 and Table 10). Aiming to scrutinize the interdependencies among the response NQLs, principal component analysis (PCA) is applied to identify a set of uncorrelated components (*j*—the principal component number, *k*—the experimental trial number, *i*—the response number, *V_ij_*—the eigenvector’s elements) [62]:

(4) $$Y_{k}=\sum_{i=1}^{p}{NQL}_{i}(k)V_{ij}$$

The principal components (Table 8, Table 9 and Table 10) present input for grey relational analysis (GRA) that transforms them into the grey relational coefficient *ε_j_* (*k*), and, finally, develops a single process performance index—the gray relational grade (*γ_k_*) [62]:

(5) $$\gamma_{k}=\sum_{j=1}^{p}\omega_{j}\varepsilon_{j}\left(k\right)$$

The ultimate process indexes (*γ_k_* ∈ [0, 1]) for different atmospheric conditions are listed in Table 8, Table 9 and Table 10. The *γ_k_* values are enumerated using the response weights from PCA (*w_j_*): (i) for the standard atmospheric conditions: 0.425, 0.189, 0.165, 0.106, 0.081, 0.020, 0.014; (ii) for the argon-rich atmosphere: 0.462, 0.216, 0.160, 0.093, 0.049, 0.018, 0.002; and (iii) for the nitrogen-rich atmosphere: 0.459, 0.185, 0.133, 0.121, 0.065, 0.026, 0.011. Therefore, all principal components are entailed in an entirely objective manner to encapsulate the total response variance. The details of the suggested process optimization procedure are given in [62], and its implementation in [26].

The optimal process variables (parameter values) are the ones that boost the process performance index. As presented in Table 8, for standard atmospheric conditions, the optimal process variables are *E_p_* = 50 and *N_p_* = 800, resulting in an excellent process index (0.9785). The settings *E_p_* = 50 and *N_p_* = 800 (process index = 0.9273) and *E_p_* = 50 and *N_p_* = 100 (process index = 0.9190) are also favorable since they produce high process performance. From Table 9, for the argon-rich atmosphere, the optimal process setting is *E_p_* = 250 and *N_p_* = 100, delivering a good process index (0.8910). The setting *E_p_* = 170 and *N_p_* = 800 is also favorable for reaching satisfactory process performance (process index = 0.8766). For the nitrogen-rich atmosphere (Table 10) the optimal process setting is *E_p_* = 170 and *N_p_* = 800, producing good process performance (process index = 0.8949). In addition, the setting *E_p_* = 250 and *N_p_* = 800 is also acceptable in terms of generating relatively good process performance (process index = 0.8565). Overall, it is evident that the optimal setting for the standard atmosphere (air) is the most beneficial since it achieves a very high process performance, while the optimal parameters for the argon- and nitrogen-rich atmospheres provide similar, satisfactorily high levels of process performance.

## 4. Conclusions

A titanium-based Ti6Al4V alloy surface was modified by laser action in the infrared spectral region in various conditions: 10.6 μm and 1064 nm wavelengths, nanosecond (100 ns and 5 ns) and picosecond (150 ps) pulse durations, increasing numbers of accumulated pulses at different values of pulse energy and different ambient conditions: standard air and argon-rich and nitrogen-rich atmospheres. Irradiation of the Ti6Al4V alloy by a TEA CO_2_ laser beam at a 100 ns pulse duration, in all three ambient conditions, led to pronounced morphological changes on the surface: increased surface roughness, microcracks, partial formation of granular structures and formation of LIPSS, which is a novelty regarding nanosecond modification with multi-mode laser beams. Surface features on Ti6Al4V after interaction with a nanosecond Gaussian laser beam, a Nd:YAG laser with a 5 ns pulse duration, imply the ablation process is more pronounced, as crater formation is evident after applying a larger number of pulses in all three ambient conditions. In general, the lowest average ablation depth values are obtained in a nitrogen-rich atmosphere. Picosecond laser irradiation of the Ti6Al4V alloy was characterized by crater formation and LIPSS located at the periphery of the modified target at pulse energy values of 6 and 15 mJ in all three ambient conditions. The most pronounced LIPSS structures are formed in a nitrogen-rich atmosphere after 200 accumulated pulses at 5.4 J cm^−2^ fluence. It was noted that the highest ablation depth value achieved, about 36 μm, is obtained after 200 accumulated pulses at 6.1 J cm^−2^ fluence in standard laboratory conditions. The results of the chemical composition investigation imply that titanium nitride is probably formed after laser irradiation in a nitrogen-rich atmosphere under specific experimental conditions. Optimization of the laser parameters was performed for the Nd:YAG laser with a 5 ns interaction with the alloy surface. The optimum setting for a standard atmosphere (air) is the most useful as it achieves very high process performance, while the optimum parameters for an argon-rich atmosphere and for a nitrogen-rich atmosphere give a similar, satisfactory high, level of process performance. The most accurate and precise modification is achieved when laser/material interaction is performed in an argon-rich atmosphere.

## Figures and Tables

**Table 1:** The conditions and parameters of TEA CO_2_ nanosecond, Nd:YAG nanosecond and Nd:YAG picosecond laser irradiation in the interaction with Ti6Al4V alloy.

Laser	Nanosecond TEA CO_2_	Nanosecond Nd:YAG	Picosecond Nd:YAG
Wavelength	10.6 μm	1064 nm	1064 nm
Focal length (mm) *	135	100	170
Pulse duration	~100 ns	~5 ns	~150 ps
Number of accumulated pulses, (*N_p_*)	50, 100, 400, 800, 2000	1, 5, 10, 100, 800, 2000	10, 50, 100, 150, 200, 400
Pulse energy, *E_p_* (mJ)	155 ± 5 175 ± 5	50 ± 3 170 ± 5 250 ± 10	6.0 ± 0.5 15 ± 2 30 ± 4
Environmental conditions	Standard atmospheric conditions (SA) Nitrogen-rich atmosphere (NA) Argon-rich atmosphere (AA)		
Fluence, *F* (J cm^−2^)	SA: 3.9; 5.3 NA: 5.5; 6.2 AA: 4.7; 6.0	SA: 19.1; 64.9; 95.5 NA: 16.3; 55.5, 81.7 AA: 17.0; 57.7; 84.9	SA: 6.1; 15.2; 30.5 NA: 5.4; 13.6; 27.2 AA: 9.4; 23.5; 47.1

* The stand-off distance, i.e., the distance between the sample and lens, is the same as the focal length with all lasers, to achieve the most effective ablation.

**Table 2:** Damage threshold fluences, *F_th_* (J cm^−2^), for Ti6Al4V.

Environmental Conditions	*F_th_* (J cm^−2^)		
	Nd:YAG (150 ps)	Nd:YAG (5 ns)	TEA CO_2_ (100 ns)
Standard atmosphere (SA)	2.13	3.09	3.92
Nitrogen-rich atmosphere (NA)	1.13	9.31	5.54
Argon-rich atmosphere (AA)	1.21	14.46	4.65
HAZ (nm)	~18	~105	~469

**Table 3:** EDS analysis of the Ti6Al4V surface before and after nanosecond laser irradiation in different experimental conditions.

Environmental Conditions	Irradiation Conditions	Location	N	O	wt% Al	Ti	V
	Unmodified				5.6 ± 0.26	90.9 ± 0.78	3.5 ± 0.22
SA	F = 6.2 J cm^−2^	center		10.4 ± 0.81	4.8 ± 0.13	82.2 ± 0.74	2.6 ± 0.21
	*N_p_* = 100						
NA	F = 6.2 J cm^−2^	center		34.2 ± 0.94	3.6 ± 0.13	59.5 ± 0.88	2.7 ± 0.19
	*N_p_* = 400	periphery		38.9 ± 0.91	3.8 ± 0.12	55.3 ± 0.85	2.0 ± 0.19
	F = 6.2 J cm^−2^	center	12.6 ± 0.45	10.0 ± 0.64	3.7 ± 0.15	71.0 ± 0.76	2.7 ± 0.29
	*N_p_* = 2000	periphery	9.9 ± 0.38	11.1 ± 0.68	3.1 ± 0.15	72.7 ± 0.76	3.2 ± 0.33
AA	F = 6.2 J cm^−2^	center		35.0 ± 0.91	3.6 ± 0.18	58.6 ± 0.69	2.8 ± 0.36
	*N_p_* = 400	periphery		38.1 ± 0.98	3.4 ± 0.14	56.2 ± 0.65	2.3 ± 0.22

**Table 4:** EDS analysis of the Ti6Al4V surface before and after picosecond laser irradiation in different experimental conditions.

Environmental Conditions	Irradiation Conditions	Location	N	O	wt% Al	Ti	V
	Unmodified				5.6 ± 0.25	90.9 ± 0.85	3.5 ± 0.38
SA	F = 15.2 J cm^−2^	center		18.3 ± 0.64	4.0 ± 0.41	74.7 ± 0.82	3.0 ± 0.33
	*N_p_* = 200	periphery		29.8 ± 0.85	4.0 ± 0.42	63.5 ± 0.74	2.7 ± 0.0.28
NA	F = 13.6 J cm^−2^ *N_p_* = 100	center periphery	13.7 ± 0.44 10.6 ± 0.40	10.0 ± 0.74 18.4 ± 0.66	3.7 ± 0.39 3.9 ± 0.44	69.9 ± 0.75 64.4 ± 0.69	2.7 ± 0.28 2.7 ± 0.29
AA	F = 9.4 J cm^−2^ *N_p_* = 400	center periphery		16.3 ± 0.58 15.7 ± 0.57	4.1 ± 0.48 4.7 ± 0.51	76.5 ± 0.72 76.4 ± 0.72	3.1 ± 0.31 3.2 ± 0.35

**Table 5:** Experimental results for standard atmospheric conditions.

Exp. Trial No.	Process Variables		Responses						
	*E_p_* (mJ)	*N_p_*	*R_a_* (μm)	*rms* (μm)	*PV* (μm)	*Kr*	*DA* (μm)	*VA* (μm pulse^−1^)	*C*
1	50	1	1.23	6.52	0.22	0	0.71	0.710	0.89
2	50	5	1.66	13.71	0.95	0	1.02	0.204	0.89
3	50	10	1.15	59.60	1.65	0	1.68	0.168	0.90
4	50	100	3.11	27.09	4.17	1	8.12	0.081	0.91
5	50	800	6.51	67.59	8.05	1	15.69	0.020	0.93
6	50	2000	13.08	65.58	12.73	1	30.35	0.015	0.92
7	170	1	0.93	35.89	1.15	0	1.29	1.290	0.84
8	170	5	1.01	19.56	1.35	0	2.06	0.412	0.84
9	170	10	1.48	23.41	2.02	0	3.69	0.369	0.90
10	170	100	2.27	23.62	2.89	0	4.91	0.049	0.94
11	170	800	4.42	40.22	5.14	0	13.46	0.017	0.91
12	170	2000	7.84	154.95	9.11	0	20.44	0.010	0.90
13	250	1	1.64	8.21	0.21	0	0.66	0.660	0.86
14	250	5	1.87	54.04	0.21	0	1.07	0.214	0.86
15	250	10	2.29	25.34	0.19	0	3.23	0.323	0.85
16	250	100	5.15	65.24	0.33	1	17.23	0.172	0.80
17	250	800	5.00	116.92	4.72	1	21.19	0.026	0.80
18	250	2000	6.30	113.94	8.03	1	32.27	0.016	0.80

**Table 6:** Experimental results for an argon-rich atmosphere.

Exp. Trial No.	Process Variables		Responses						
	*E_p_* (mJ)	*N_p_*	*R_a_* (μm)	*rms* (μm)	*PV* (μm)	*Kr*	*DA* (μm)	*VA* (μm pulse^−1^)	*C*
1	50	1	0.35	4.42	0.20	0	2.37	2.370	0.93
2	50	5	0.59	5.81	0.17	0	3.98	0.796	0.93
3	50	10	0.99	7.56	0.20	0	8.97	0.897	0.96
4	50	100	5.83	43.89	0.64	0	14.84	0.148	0.96
5	50	800	6.18	136.43	5.95	1	67.96	0.085	0.86
6	50	2000	6.81	92.42	13.97	1	67.01	0.034	0.90
7	170	1	0.27	3.85	1.15	0	1.12	1.120	0.93
8	170	5	0.47	20.40	0.78	0	1.22	0.244	0.93
9	170	10	0.81	23.11	1.31	0	2.62	0.262	0.97
10	170	100	6.64	68.05	9.14	0	27.93	0.279	0.94
11	170	800	20.40	145.76	21.02	1	51.01	0.064	0.95
12	170	2000	23.40	158.31	36.98	1	63.42	0.032	0.88
13	250	1	0.52	37.85	4.11	0	1.06	1.060	0.88
14	250	5	0.89	25.37	0.89	0	1.27	0.254	0.88
15	250	10	3.03	28.71	1.51	0	2.02	0.202	0.93
16	250	100	6.27	63.22	8.92	1	20.74	0.207	0.93
17	250	800	12.90	107.92	17.02	1	63.01	0.079	0.93
18	250	2000	32.80	219.95	35.81	1	67.61	0.034	0.91

**Table 7:** Experimental results for a nitrogen-rich atmosphere.

Exp. Trial No.	Process Variables		Responses						
	*E_p_* (mJ)	*N_p_*	*Ra* (μm)	*rms* (μm)	*PV* (μm)	*Kr*	*DA* (μm)	*VA* (μm pulse^−1^)	*C*
1	50	1	0.57	14.48	0.20	0	0.21	0.210	0.83
2	50	5	0.67	9.65	0.21	0	2.30	0.460	0.83
3	50	10	1.25	30.22	0.20	0	4.64	0.464	0.91
4	50	100	2.97	44.51	0.22	0	11.62	0.116	0.90
5	50	800	8.48	83.97	8.32	1	49.47	0.062	0.79
6	50	2000	13.40	92.39	17.63	1	65.98	0.033	0.82
7	170	1	1.44	8.43	0.23	0	0.54	0.540	0.86
8	170	5	2.25	21.43	0.25	0	1.81	0.362	0.86
9	170	10	2.88	11.44	0.19	0	6.45	0.645	0.91
10	170	100	2.71	20.04	0.21	0	12.56	0.126	0.94
11	170	800	4.27	144.18	7.08	1	33.92	0.042	0.88
12	170	2000	7.05	136.94	10.01	1	38.73	0.019	0.90
13	250	1	0.27	9.03	0.15	0	1.04	1.040	0.87
14	250	5	0.79	19.53	0.16	0	2.09	0.418	0.87
15	250	10	1.24	28.38	0.18	0	3.57	0.357	0.86
16	250	100	2.33	21.81	0.23	1	11.78	0.118	0.81
17	250	800	3.67	122.02	4.58	1	16.69	0.021	0.92
18	250	2000	7.55	136.01	8.41	1	29.39	0.015	0.92

**Table 8:** Results of experimental data processing for standard atmospheric conditions.

Exp. Trial No.	Normalized Quality Losses (NQLs)							Principal Component Scores							Process Index
	NQL *Ra*	NQL *rms*	NQL *PV*	NQL *Kr*	NQL *DA*	NQL *VA*	NQL *C*	Y1	Y2	Y3	Y4	Y5	Y6	Y7	
1	0.570	1.000	0.754	1.000	0.864	0.000	0.302	1.803	−0.094	0.159	−0.616	−0.061	−0.141	0.202	0.5697
2	0.310	0.225	0.040	1.000	0.418	0.001	0.302	0.816	−0.307	−0.258	−0.586	0.062	−0.490	−0.010	0.5898
3	0.652	0.010	0.013	1.000	0.154	0.002	0.254	0.679	−0.432	−0.608	−0.648	0.141	−0.234	0.045	0.5420
4	0.085	0.056	0.002	0.000	0.006	0.008	0.175	0.057	0.107	−0.074	−0.111	−0.079	−0.004	0.047	0.9190
5	0.015	0.008	0.000	0.000	0.001	0.141	0.055	−0.032	−0.010	0.045	−0.126	−0.057	0.030	0.004	0.9785
6	0.000	0.008	0.000	0.000	0.000	0.254	0.114	−0.066	−0.004	0.093	−0.232	−0.096	0.036	0.007	0.9273
7	1.000	0.031	0.027	1.000	0.261	0.000	0.665	0.869	−0.197	−0.902	−0.935	−0.048	−0.166	0.017	0.5403
8	0.848	0.110	0.019	1.000	0.102	0.000	0.665	0.767	−0.176	−0.814	−0.900	−0.002	−0.237	0.184	0.5628
9	0.392	0.076	0.009	1.000	0.032	0.000	0.237	0.556	−0.400	−0.441	−0.585	0.215	−0.365	0.175	0.5706
10	0.164	0.075	0.004	1.000	0.018	0.023	0.000	0.463	−0.534	−0.228	−0.427	0.309	−0.421	0.162	0.6084
11	0.039	0.025	0.001	1.000	0.002	0.196	0.175	0.339	−0.447	−0.128	−0.629	0.289	−0.488	0.149	0.6567
12	0.009	0.000	0.000	1.000	0.001	1.000	0.237	0.097	−0.665	0.243	−1.197	0.065	−0.305	0.113	0.6990
13	0.318	0.630	0.829	1.000	1.000	0.000	0.513	1.638	0.126	0.210	−0.761	0.146	−0.296	−0.119	0.5393
14	0.244	0.013	0.829	1.000	0.380	0.001	0.513	0.986	0.096	−0.078	−0.853	0.633	−0.182	−0.057	0.6084
15	0.161	0.065	1.000	1.000	0.041	0.000	0.585	0.873	0.201	−0.044	−0.904	0.822	−0.116	0.244	0.5976
16	0.028	0.008	0.330	0.000	0.001	0.002	1.000	0.140	0.807	−0.147	−0.628	0.026	−0.107	0.107	0.7245
17	0.030	0.001	0.001	0.000	0.001	0.113	1.000	−0.025	0.666	−0.209	−0.651	−0.202	−0.233	0.080	0.7419
18	0.017	0.002	0.000	0.000	0.000	0.245	1.000	−0.066	0.624	−0.139	−0.736	−0.238	−0.206	0.076	0.7359

**Table 9:** Results of experimental data processing for an argon-rich atmosphere.

Exp. Trial No.	Normalized Quality Losses (NQLs)							Principal Component Scores							Process Index
	NQL *Ra*	NQL *rms*	NQL *PV*	NQL *Kr*	NQL *DA*	NQL *VA*	NQL *C*	Y1	Y2	Y3	Y4	Y5	Y6	Y7	
1	0.613	0.759	0.717	1.000	0.206	0.000	0.303	1.294	−0.068	−0.457	−0.566	0.444	0.440	0.040	0.5164
2	0.220	0.439	1.000	1.000	0.071	0.001	0.303	1.001	0.247	−0.282	−0.898	0.545	0.305	0.025	0.5080
3	0.075	0.259	0.717	1.000	0.014	0.001	0.037	0.805	0.340	−0.008	−0.611	0.590	0.323	0.008	0.5998
4	0.002	0.007	0.069	1.000	0.005	0.044	0.087	0.443	0.025	0.325	−0.293	0.574	0.541	0.054	0.6903
5	0.002	0.000	0.001	0.000	0.000	0.134	1.000	−0.246	−0.600	−0.266	−0.649	−0.098	0.306	0.055	0.6573
6	0.001	0.001	0.000	0.000	0.000	1.000	0.557	−0.489	−0.443	−0.635	−0.214	0.625	0.189	0.019	0.5685
7	1.000	1.000	0.021	1.000	0.894	0.001	0.283	1.615	−0.893	−0.332	0.020	0.441	0.392	−0.102	0.4383
8	0.324	0.035	0.046	1.000	0.753	0.016	0.283	0.818	−0.625	0.347	−0.324	0.659	0.178	0.060	0.4977
9	0.110	0.027	0.016	1.000	0.171	0.014	0.000	0.571	−0.073	0.364	−0.175	0.589	0.445	0.061	0.6484
10	0.002	0.003	0.000	1.000	0.001	0.012	0.259	0.398	−0.102	0.332	−0.372	0.508	0.623	0.060	0.6693
11	0.000	0.000	0.000	0.000	0.000	0.239	0.129	−0.116	−0.103	−0.151	−0.048	0.150	0.044	0.004	0.8766
12	0.000	0.000	0.000	0.000	0.000	0.956	0.759	−0.513	−0.555	−0.650	−0.356	0.554	0.249	0.031	0.5368
13	0.267	0.010	0.002	1.000	1.000	0.001	0.795	0.754	−1.076	0.354	−0.663	0.611	0.197	−0.010	0.4622
14	0.091	0.023	0.035	1.000	0.696	0.015	0.795	0.581	−0.835	0.324	−0.728	0.566	0.365	−0.054	0.5064
15	0.008	0.018	0.012	1.000	0.280	0.023	0.313	0.488	−0.297	0.362	−0.411	0.571	0.473	−0.020	0.5936
16	0.002	0.003	0.000	0.000	0.002	0.022	0.312	−0.066	−0.187	−0.075	−0.205	−0.045	0.095	0.016	0.8910
17	0.000	0.001	0.000	0.000	0.000	0.154	0.343	−0.125	−0.218	−0.149	−0.205	0.046	0.107	0.017	0.8289
18	0.000	0.000	0.000	0.000	0.000	0.856	0.464	−0.417	−0.371	−0.540	−0.175	0.538	0.158	0.016	0.6105

**Table 10:** Results of experimental data processing for a nitrogen-rich atmosphere.

Exp. Trial No.	Normalized Quality Losses (NQLs)							Principal Component Scores							Process Index
	NQL *Ra*	NQL *rms*	NQL *PV*	NQL *Kr*	NQL *DA*	NQL *VA*	NQL *C*	Y1	Y2	Y3	Y4	Y5	Y6	Y7	
1	0.222	0.336	0.568	1.000	1.000	0.004	0.653	1.212	0.602	0.178	−0.713	0.022	−0.650	0.340	0.4691
2	0.161	0.762	0.501	1.000	0.008	0.001	0.653	1.154	0.190	−0.028	0.162	0.451	−0.733	0.386	0.5817
3	0.046	0.075	0.568	1.000	0.002	0.001	0.196	0.827	−0.187	−0.294	−0.164	−0.061	−0.629	0.371	0.5857
4	0.008	0.033	0.480	1.000	0.000	0.014	0.205	0.745	−0.166	−0.316	−0.183	−0.045	−0.613	0.424	0.5993
5	0.001	0.007	0.000	0.000	0.000	0.051	1.000	0.037	0.800	−0.022	0.329	−0.010	−0.475	0.165	0.7956
6	0.000	0.005	0.000	0.000	0.000	0.186	0.747	−0.031	0.561	0.067	0.227	0.006	−0.451	0.137	0.8085
7	0.034	1.000	0.412	1.000	0.159	0.000	0.464	1.181	0.121	0.030	−0.015	0.730	−0.609	0.311	0.6228
8	0.014	0.152	0.348	1.000	0.013	0.001	0.464	0.753	0.077	−0.281	−0.079	0.087	−0.649	0.521	0.6395
9	0.008	0.541	0.605	1.000	0.001	0.000	0.169	1.028	−0.218	−0.178	−0.087	0.318	−0.618	0.244	0.5665
10	0.009	0.174	0.501	1.000	0.000	0.012	0.000	0.806	−0.337	−0.264	−0.221	0.064	−0.523	0.351	0.5601
11	0.004	0.000	0.000	0.000	0.000	0.112	0.322	−0.025	0.234	0.048	0.095	0.002	−0.214	0.063	0.8949
12	0.001	0.000	0.000	0.000	0.000	0.540	0.202	−0.206	0.029	0.297	0.000	0.045	−0.438	0.080	0.8236
13	1.000	0.872	1.000	1.000	0.040	0.000	0.403	1.751	−0.128	0.510	0.347	−0.020	−0.625	0.267	0.4816
14	0.116	0.183	0.875	1.000	0.010	0.001	0.403	1.067	−0.075	−0.255	−0.054	−0.118	−0.837	0.218	0.6059
15	0.047	0.085	0.687	1.000	0.003	0.001	0.493	0.909	0.033	−0.317	−0.062	−0.101	−0.815	0.345	0.6305
16	0.013	0.146	0.417	0.000	0.000	0.014	0.795	0.318	0.565	−0.022	0.301	−0.049	−0.541	−0.146	0.6964
17	0.005	0.001	0.001	0.000	0.000	0.447	0.134	−0.170	−0.004	0.251	−0.010	0.036	−0.348	0.062	0.8565
18	0.001	0.000	0.000	0.000	0.000	1.000	0.140	−0.395	−0.136	0.562	−0.078	0.088	−0.709	0.111	0.6754

## Data Availability

The data presented in this study are available on request from the corresponding author. The data are not publicly available due to institutional policies.
